# Effect of Dietary Tryptophan on Growth, Intestinal Microbiota, and Intestinal Gene Expression in an Improved Triploid Crucian Carp

**DOI:** 10.3389/fnut.2021.676035

**Published:** 2021-06-17

**Authors:** Yawei Fu, Xiaoxiao Liang, Donghua Li, Hu Gao, Yadong Wang, Wenting Li, Kang Xu, Fangzhou Hu

**Affiliations:** ^1^CAS Key Laboratory of Agro-Ecological Processes in Subtropical Region, Hunan Provincial Key Laboratory of Animal Nutritional Physiology and Metabolic Process, National Engineering Laboratory for Pollution Control and Waste Utilization in Livestock and Poultry Production, Institute of Subtropical Agriculture, Chinese Academy of Sciences, Changsha, China; ^2^College of Animal Science and Technology, Henan Agricultural University, Zhengzhou, China; ^3^State Key Laboratory of Developmental Biology of Freshwater Fish, College of Life Sciences, Hunan Normal University, Changsha, China

**Keywords:** tryptophan, improved triploid crucian carp, transcriptome, intestinal flora, 16S rRNA

## Abstract

Tryptophan (Trp) has received increasing attention in the maintenance of intestinal function. In this study, improved triploid crucian carp (ITCC) fed diets containing 6.35 g kg^−1^ Trp had higher average daily gain (ADG) and improved villus height (VH) and crypt depth (CD) in the intestine compared to the control group. To elucidate the potential mechanisms, we used RNA sequencing (RNA-seq) to investigate changes in the intestinal transcriptome and 16S rRNA gene sequencing to measure the intestinal microbiota in response to 6.35 g kg^−1^ Trp feeding in ITCC. Dietary Trp altered intestinal gene expression involved in nutrient transport and metabolism. Differentially expressed transcripts (DETs) were highly enriched in key pathways containing protein digestion and absorption and the AMPK signaling pathway. 16S rRNA sequencing showed that 6.35 g kg^−1^ Trp significantly increased the abundance of the genus *Cetobacterium*, and the Firmicutes/Bacteroidetes ratio at the phylum level (*P* < 0.05). In addition, bacterial richness indices (Simpson index) significantly increased (*P* < 0.05) community evenness in response to 6.35 g kg^−1^ Trp. In conclusion, appropriate dietary Trp improves the growth performance, and influences the intestinal flora of ITCC. This study might be helpful to guide the supply of dietary exogenous Trp in ITCC breeding.

## Introduction

Improving the growth rate and feed conversion rate of fish is essential for the sustainable development of aquaculture (1, 2). Amino acids play important roles in the nutrient metabolism of cultured fishes. Dietary nutrients affect gut microbial diversity and composition (3). Numerous studies describe the link between Trp metabolism and fish health (4). Trp cannot be synthesized exogenously in fish, and must be obtained via food (5, 6). Appropriate feeding of Trp has positive effects on the growth performance and intestinal health status of fish by regulating intestinal immune tolerance, maintaining microbial homeostasis, and inhibiting inflammation (5, 6). Besides, there is growing evidence that Trp deficiency could cause negative impacts on growth performance in fish (7–9). For example, fish growth performance and the structural integrity of the intestines were altered in a Trp deficient scenario, which translated into lower disease resistance (6, 10). Similarly, Trp deficiency causes depressed growth and efficiency of feed conversion and low protein retention, as reported for other fish species (11–14).

The microbial balance of the intestinal flora is associated with the gut health, which can be affected by dietary constituents and commensal bacteria (15, 16). The intestinal flora could affect food digestion and absorption, and nutrients can also affect the composition of intestinal microbes (17, 18). Previous studies have demonstrated that Trp could alter the intestinal microbial composition and diversity (19). The metabolism of Trp by the intestinal microbiome could also affect intestinal homeostasis (20, 21). Trp catabolites are essential signaling molecules in microbial communities, and important mediators for regulating a diverse array of physiological systems in fish (4). Gut microbes are primary participants in Trp metabolism; it is estimated that 90% of serotonin in the human body is produced by gut microbes (22).

In our previous studies, the ITCC was produced by crossing improved tetraploid males with improved diploid female red crucian carp, which has excellent traits of fast growth rate and sterility (23). With the expansion of improved triploid crucian carp farming, the breed of ITCC and research on the development of its feeding should be developed. However, information regarding the effects of amino acids on the growth performance and intestinal health status of ITCC is lacking. Moreover, little information has been done on the effect of Trp on the growth performance and intestinal health of ITCC. In this study, we aimed to explore the effects of Trp supplementation on growth, intestinal microbiota, and intestinal gene expression in ITCC. This study will provide guidance for developing effective nutritional strategies and feeding practices to improve ITCC health.

## Materials and Methods

### Ethics Statement

All experimental animals used in this study were treated humanely, following the Animal Welfare Committee of the Institute of Subtropical Agriculture (201703-64C), Chinese Academy of Sciences, Changsha, China.

### Experimental Animals and Tissue Samples

A total of 450 healthy ITCC and weight-matched ITCC (23) were randomly divided into five groups, with three biological replicates of each group, and 30 fishes per biological replicate. The Trp concentrations in the five experimental diets were determined to be 1.85 (control), 3.35, 4.85, 6.35, and 7.85 g kg^−1^ Trp diet (basal diets supplemented with 0, 1.5, 3.0, 4.5, and 6.0 g kg^−1^ Trp). The fishes were weighed at the beginning and end of the 4-week feeding trial.

### Sample Collections

At the end of the feeding trial, all the experimental fish fasted for 12 h. Three fish from each replicate (a total of 45 fish) were randomly selected, anesthetized, sacrificed, and sampled according to the method described in a previous study (24). For other biochemical parameters and molecular analysis, the intestinal tract and distal intestinal contents were quickly removed, frozen in liquid nitrogen, and stored at −80°C until use. Meanwhile, we collected section samples from each group and fixed them in a 4% paraformaldehyde solution for histologic analysis (25).

### RNA Extraction

Transcriptome sequencing was performed on a total of 6 samples from the 6.35 Trp group (6.35 g kg^−1^ Trp diet group) and the 1.85 Trp group (control group). Total RNA was isolated from the intestinal tract of fish using an RNAiso Plus kit (Takara, Kyoto, Japan). Total RNA was purified using a TruSeq RNA Sample Prep Kit 52 (New England Biolabs, Ipswich, MA, USA). RNA degradation and contamination were detected using 1% agarose gels. The purity of the total RNA was assessed with a NanoPhotometer® spectrophotometer (IMPLEN, CA, USA). The total RNA concentration was measured using a Qubit® RNA Assay Kit in Qubit® 2.0 Fluorometer (Life Technologies, CA, USA). The integrity of the total RNA was estimated using an RNA Nano 6000 Assay Kit of the Bioanalyzer 2100 system (Agilent Technologies, CA, USA).

### Library Preparation for Transcriptome Sequencing

Three samples were pooled to make one biological replicate and experiment was done using three technical replicates. A total amount of 1.5 μg RNA per sample was used as input material for the RNA sample preparations. Sequencing libraries were generated using the NEBNext® Ultra™ RNA Library Prep Kit of Illumina® (NEB, USA) following the manufacturer's protocols, and index codes were added to attribute sequences to each sample. Briefly, mRNA was purified from total RNA using poly-T oligo-attached magnetic beads. Fragmentation was carried out using divalent cations under elevated temperature in NEBNext First Strand Synthesis Reaction Buffer (5X). First-strand cDNA was synthesized using random hexamer primers and M-MuL5 Reverse Transcriptase (RNase H). Second strand cDNA synthesis was subsequently performed using DNA Polymerase I and RNase H. The remaining overhangs were converted into blunt ends via exonuclease/polymerase activities. After adenylation of the 3′ ends of DNA fragments, NEBNext adaptor with a hairpin loop structure was ligated to prepare for hybridization. To preferentially select cDNA fragments 250–300 bp in length, the library fragments were purified with the AMPure XP system (Beckman Coulter, Beverly, USA). Then, 3 μl of USER Enzyme (NEB, USA) was used with size-selected, adaptor-ligated cDNA at 37°C for 15 min followed by 5 min at 95°C before PCR. Then PCR was performed with Phusion High-Fidelity DNA polymerase, universal PCR primers, and Index (X) Primer. Finally, PCR products were purified (AMPure XP system), and library quality was assessed on the Agilent Bioanalyzer 2100 system.

### RNA-Seq Data Analysis

Clustering of the index-coded samples was performed on a cBot Cluster Generation System using TruSeq PE Cluster Kit 53-cBot-HS (Illumina) according to the manufacturer's instructions. Differential expression analysis of two groups (three biological replicates per group) was performed using the DESeq R package (1.10.1) (26). DESeq provides statistical routines for determining differential expression in digital gene expression data using a model based on the negative binomial distribution. The resulting *P*-values were adjusted using Benjamini and Hochberg's approach to control the false discovery rate. Genes with an adjusted *P* < 0.05 found by DESeq were assigned as differentially expressed. To obtain significantly different genes, we set the screening criteria as *P*-value (padj) ≤ 0.001 and difference multiple |FoldChange| ≥ 2. Cluster analysis was used to cluster genes with the same or similar expression patterns, which might have similar functions or participate in the same biological process. Cluster analysis of a heat-map for DEGs was performed by the pheatmap R package (27).

Gene function was annotated based on the following databases: Nr (NCBI non-redundant protein sequence, ftp://ftp.ncbi.nih.gov/blast/db/); Nt (NCBI non-redundant nucleotide sequence); Pfam (Protein family http://pfam.xfam.org/); KOG/COG (Clusters of Orthologous Groups of proteins, http://www.ncbi.nlm.nih.gov/KOG/); Swiss-Prot (a manually annotated and reviewed sequence database http://www.uniprot.org/); KO (KEGG Ortholog database, http://ccb.jhu.edu/software/tophat/index.shtml); GO (Gene Ontology, and STRING database. The Protein–protein interaction networks (PPIs) information of these DEGs were predicted by STRING database. After mapping the DEGs into this database, and a combined score ≥0.4 were exported. Then, the PPIs of these SDEGs were visualized in Cytoscape, and the hub genes among the PPI network were identified and ranked using CytoHubba plugin and the maximal clique centrality (MCC) method of Cytoscape software (28).

### Real-Time PCR Analysis

We randomly selected 8 genes (including four upregulated genes and four downregulated genes) for real-time PCR in 1.85Trp and 6.35Trp groups to confirm the reproducibility and accuracy of the RNA-seq gene expression data. Using an RNAiso Plus kit (Takara, Kyoto, Japan), total RNA was isolated from intestinal tract tissues of fishes. After checking the RNA quality, as described in the RNA extraction section, reverse transcription was performed using the PrimeScript™ RT Reagent Kit with gDNA Eraser (Takara, Kyoto, Japan) according to the manufacturer's protocol. Real-time PCR experiments were performed using a LightCycler® 96 Real-Time PCR system (Roche Applied Science) in a 25 μL reaction volume containing 12.5 μL of 2 × SYBR® Premix Ex TaqTM II (Tli RNaseH Plus; Takara, Kyoto, Japan), 1.25 μL each of the forward and reverse primers (10 μM), 8 μL of deionized water, and 2 μL (~100 ng) of cDNA. The β-actin gene was used as the reference gene, and the primers of eight genes were designed using Primer. The thermal cycling conditions were 3 min at 95°C, followed by 37 reaction cycles (95°C for 30 s, 60°C for 30 s, and 72°C for 30 s), and an extension for 10 min at 72°C. We calculated the relative gene expression levels with the comparative CT method (referred to as the 2^−ΔΔCT^ method) (29), with three replicates for each reaction.

### DNA Extraction, 16S rRNA Sequencing

DNA was extracted from the intestinal contents using a fecal DNA kit (Omega, USA) and then eluted in a 50 μL eluent buffer. The primers (F:5′-ACTCCTACGGGAGGCAGCAG-3′; R: 5′-GGACTACHVGGGTWT-CTAAT-3′) were used in the PCR amplification for the V3–V4 region of the bacterial 16S rRNA gene. PCR analysis was performed with 25 μL reactions containing 12.5 μL of PCR premix, 2.5 μL of each primer, 25 ng of template DNA, and PCR-grade water to equalize the final volumes. The PCR products were detected by 2% agarose gel electrophoresis and then purified using a gel recovery kit (Thermo Scientific, USA). The libraries were constructed using the Ion Plus Fragment Library Kit 48 reactions library building kit (Thermo Fisher, USA) and qualified by Qubit quantification and library assay, Single-ended sequencing was performed using Ion S5TMXL (Thermo Fisher, USA).

The clean reads of all samples were clustered using UPARSE software. The sequences were clustered into OTUs with 97% identity, and the sequences with the highest frequency of occurrence were selected as the representative sequences of OTUs. Species annotation analysis was performed using the Mothur method with the SSU rRNA database of SILVA132 for classification (threshold set between 0.8 and 1). Multiple sequence alignment was performed using MUSCLE software, and then the data were homogenized. Alpha diversity analysis and beta diversity analysis were based on the homogenized data. PCoA was plotted using R software (Version 2.15.3). The WGCNA, stats and ggplot2 were used for PCoA analysis. LEfSe was performed by LEfSe software.

### Statistical Analysis

The qRT-PCR validation data were analyzed by using SPSS 18.0 (SPSS, USA). The significance of the difference between two groups was analyzed by Student's *t*-test. Differences were considered significant if the *P* < 0.05 and *P* < 0.01 were considered extremely significant. The results are presented as the mean and standard error of the mean (SEM).

## Results

### Growth Performance and Gut Morphology

**Figure 1:**
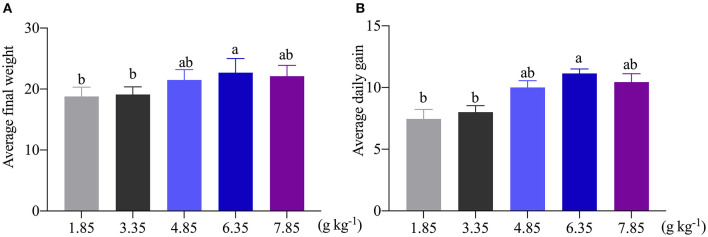
Effects of dietary tryptophan on the growth performance of ITCC. **(A)** Average final weight of ITCC. **(B)** The average daily gain of ITCC. The Trp concentrations in the five experimental diets were determined to be 1.85 (control), 3.35, 4.85, 6.35, and 7.85 g kg^−1^ Trp diet (basal diets supplemented with 0, 1.5, 3.0, 4.5, and 6.0 g kg^−1^ Trp).

As shown in Figure 1, the average final weight and the ADG were significantly (*P* < 0.05) increased as affected by dietary 6.35 g kg^−1^ Trp levels compared with the control group. In this study, compared with the control group, dietary supplementation with different doses of Trp significantly altered the VH and CD (*p* < 0.05) in ITCC, and the effect was obvious when the fish were fed a diet containing 6.35 g kg^−1^Trp, while there was no difference in the ratio of VH to CD (Table 1).

**Table 1:** The effect of tryptophan on the morphology of the intestine in ITCC.

**Item**	**1.85 Trp**	**6.35 Trp**	***p***
Villous height, μm	331.68 ± 28.32^b^	548.48 ± 82.46^a^	0.003
Crypt depth, μm	94.12 ± 12.33^b^	152.54 ± 33.28^a^	0.001
VH:CD ratio	3.59 ± 0.62	4.03 ± 0.80	0.092

*VH:CD ratio, villous height to crypt depth ratio*. ^a, b^Means without a common superscript in the same row differ (P < 0.05). *All the data were presented as mean ± SEM. They were subject to t-test*.

### RNA-Seq Analysis

To reveal the molecular regulatory mechanism, we used the pooled total RNA of the control and 6.35 g kg^−1^ Trp diet groups. The cDNA library of six intestinal tissues (1.85Trp_1, 1.85Trp _2, and 1.85Trp _3 from the control group; 6.35Trp _1, 6.35Trp _2, and 6.35Trp _3 from the group fed 6.35 g kg^−1^ Trp diets) were sequenced on an Illumina HiSeq platform, with 3.11 million reads in total being generated, of which 97.5% (3.04 million) passed the filter for clean reads. The GC contents of the clean reads were 45.61–47.29% (Supplementary Table 1). By comparing to the sequencing data of the two groups, 155,547 transcripts were identified, of which 140,907 and 143,462 transcripts were identified in the 6.35 Trp group and control group, respectively.

**Figure 2:**
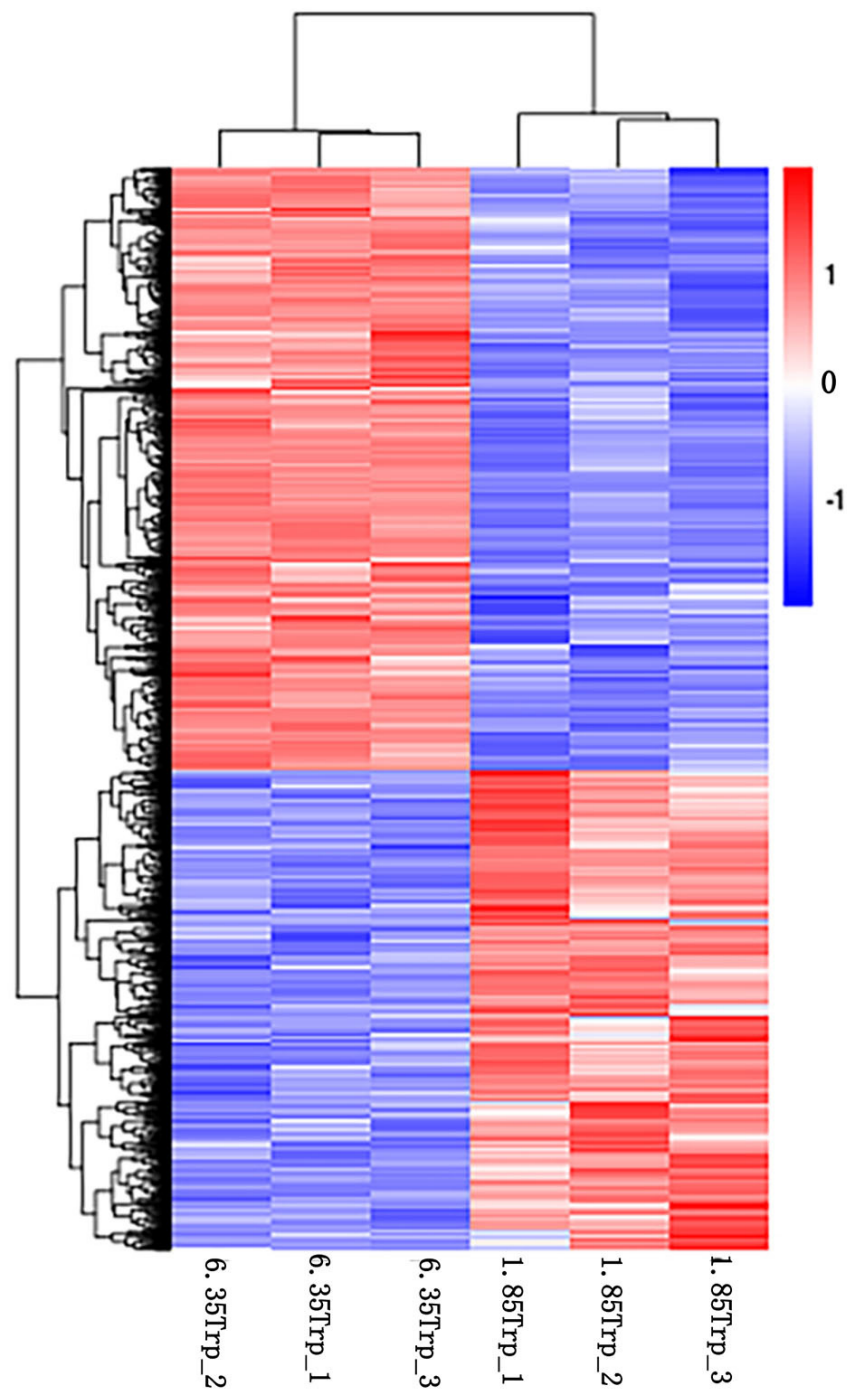
Cluster analysis of DETs by the FPKM value. The X-axis indicates the samples in the different groups. The sample on the left is from the 6.35 Trp group, and the sample on the right is from the 1.85 Trp group. The Y-axis is the gene cluster across the 1.85 and 6.35 Trp groups. Color from red to blue, indicated that the log10 (FPKM+1) values were from large to small, red color indicates high expression level and blue color indicates low expression level.

In total, we found 3,263 differentially expressed transcripts (1,443 upregulated and 1,820 downregulated transcripts) in the 6.35 Trp group compared with the control group (Supplementary Table 2). The heat map of cluster analysis of DETs showed that the gene expression patterns of DETs were clustered within groups, while the difference between the two groups was significant (Figure 2).

### GO Enrichment Analysis of DETs

**Figure 3:**
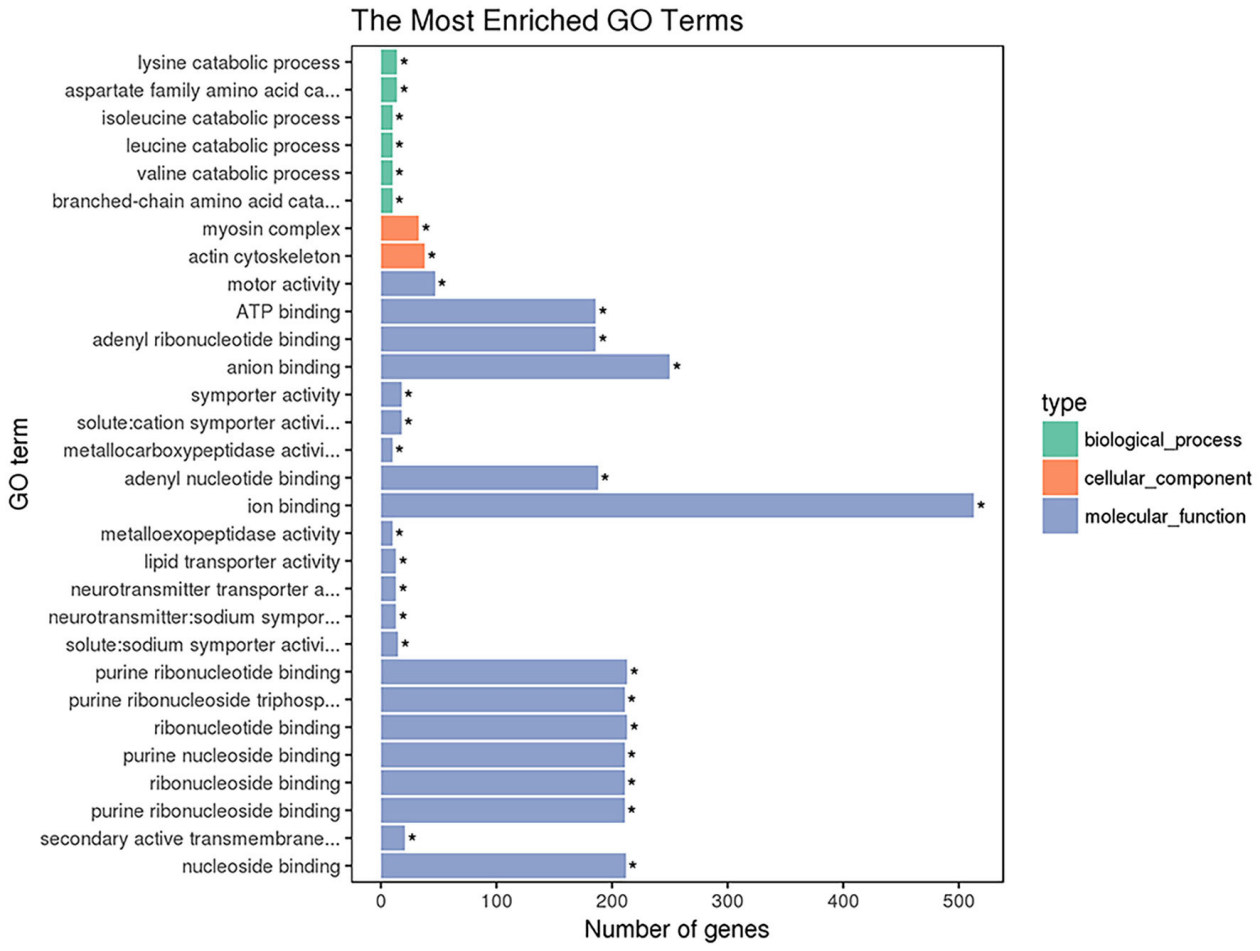
The enriched GO terms of the DETs. The X-axis indicates the number of DETs for each GO term; the y-axis corresponds to the GO terms. *represent the significantly enriched terms (*p* < 0.05).

GO enrichment analysis was performed with 1,654 DETs. A total of 2,730 GO terms were enriched, including 1,559 biological process terms, 430 cellular component terms and 741 molecular function terms (Supplementary Table 3). The top 30 most significantly enriched GO terms are shown in Figure 3. The top GO terms of the 1,164 upregulated transcripts were molecular function including motor activity and ion binding. The top GO terms of the 490 downregulated transcripts were solute: sodium symporter activity, neurotransmitter transporter activity, and neurotransmitter: sodium symporter activity. The top 22 GO terms in the molecular functions are shown in Figure 3, including motor activity, ATP binding, adenyl ribonucleotide binding, and anion binding so on. As shown in Figure 3, the top 2 GO terms belong to the cellular component category, including the myosin complex and the actin cytoskeleton.

### KEGG Pathway Analysis of DETs

The DETs were annotated into 273 KEGG pathways (Supplementary Table 4). The top 10 enriched pathways of the DETs are shown in Table 2. With regard to the KEGG pathway analysis of the upregulated genes, 11 pathways were enriched including “fatty acid biosynthesis,” “AMPK signaling pathway,” “protein digestion and absorption,” “butanoate metabolism,” and “Terpenoid backbone biosynthesis.” With regard to KEGG pathway analysis of the downregulated genes, 19 pathways were enriched, including “bile secretion,” “mineral absorption,” “renin-angiotensin system,” “cell cycle,” “carbohydrate digestion and absorption,” and “PPAR signaling pathway.”

**Table 2:** The top 10 significantly enriched KEGG pathway of DEGs.

**ID**	**KEGG term**	**Corrected *P*-value**
ko00061	Fatty acid biosynthesis	0.0000
ko04974	Protein digestion and absorption	0.0000
ko00650	Butanoate metabolism	0.0001
ko04152	AMPK signaling pathway	0.0001
ko00900	Terpenoid backbone biosynthesis	0.0001
ko04977	Vitamin digestion and absorption	0.0002
ko04973	Carbohydrate digestion and absorption	0.0011
ko00640	Propanoate metabolism	0.0013
ko04975	Fat digestion and absorption	0.0015
ko00140	Steroid hormone biosynthesis	0.0049

### Protein-Protein Interaction (PPI) Network Analysis

**Figure 4:**
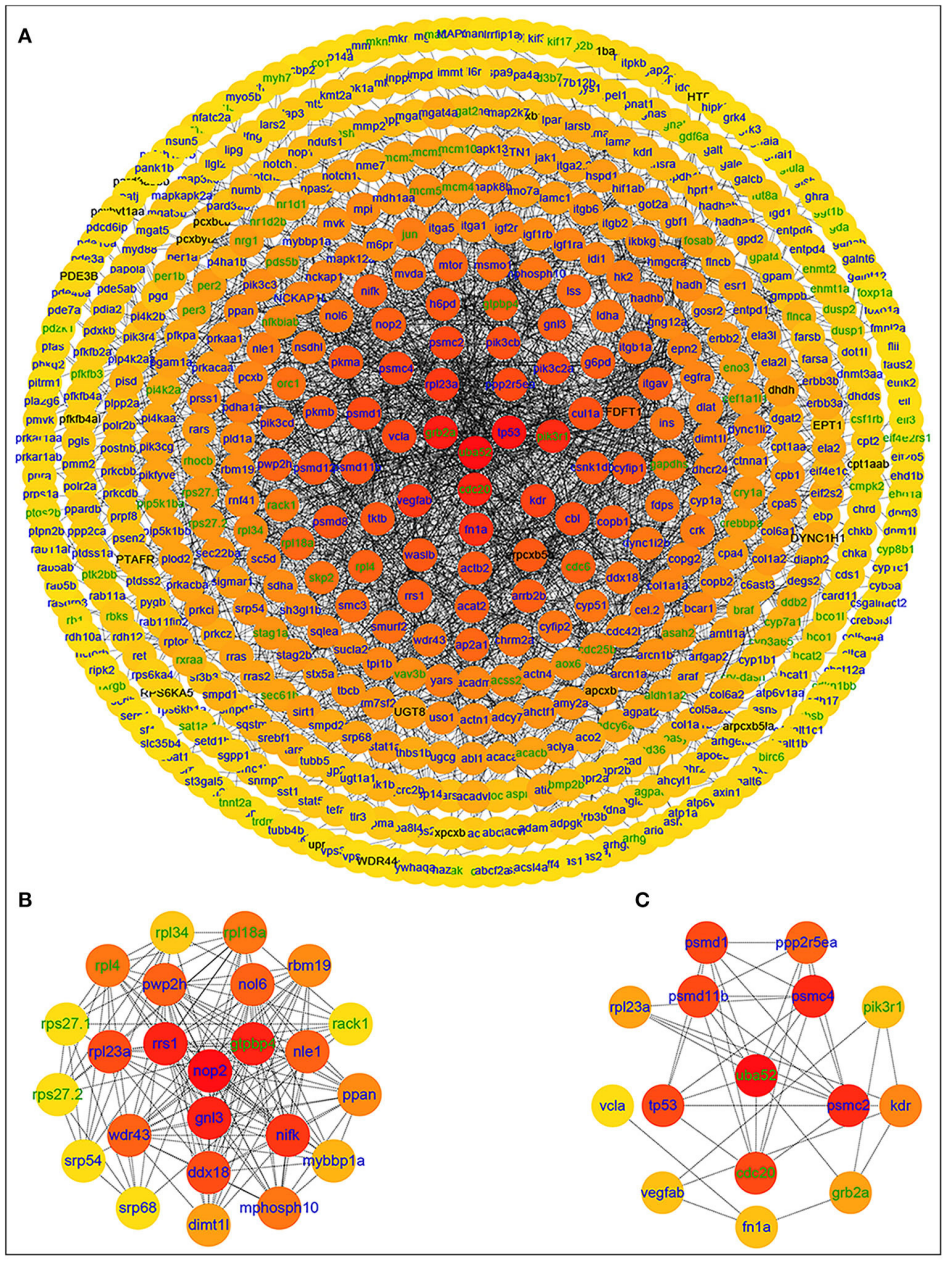
PPI network of differentially expressed genes in the group fed 6.35 g kg^−1^ Trp diets compared with the control group and two significant modules identified among the PPI network using the molecular complex detection method with a score of >13. Blue nodes represent the upregulated genes; green nodes represent the downregulated genes; **(A)** PPI network of differentially expressed genes in the group fed 6.35 g kg^−1^ Trp diets compared with the control group; **(B)** module 1, MCODE score = 23; **(C)** protein-protein interaction network of 15 hub genes.

After importing the PPI network of DETs, Cytoscape displayed modules in the default MCODE settings. Genes in these modules were then assembled for enrichment analysis using DAVID. Among them, the “metabolic pathways” was identified as the most significant pathway. Twenty-nine significant pathways were enriched in KEGG pathways. Based on the STRING database, the PPI network of 566 nodes and 2,154 protein pairs was obtained with a combined score >0.7 (Supplementary Table 5). In total, one module (module 1) with a score >13 was detected by MCODE. The hub gene ubiquitin A-52 residue ribosomal protein fusion product 1 (*UBA52*) (padj = 0.01911, log_2_FC = −2.4374) was identified with Cytohubba and MCODE (Supplementary Table 6; Figure 4).

### Real-Time PCR Validation

**Figure 5:**
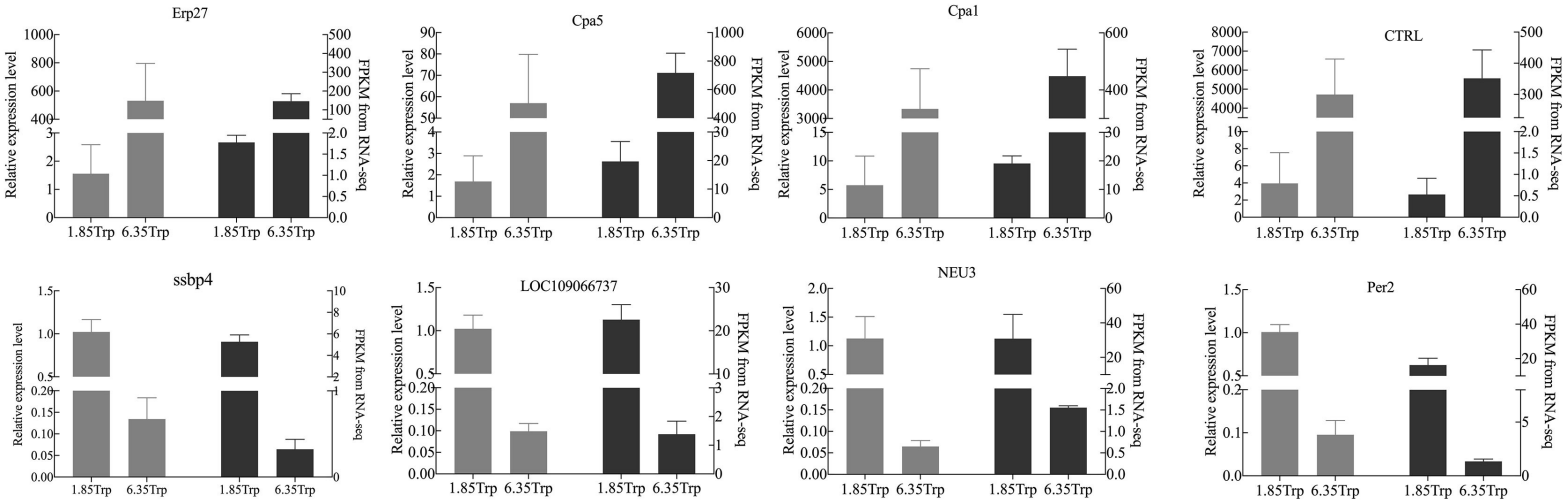
Comparison of the gene expression levels of RNA-seq with real-time PCR. The right axis represents the expression levels determined by RNA-seq in FPKM units, and the left axis represents gene expression levels determined by real-time PCR. Bars represent the mean (±SE) of three samples. The black column indicates the FPKM value; the grey column indicates the real-time PCR using β-actin as a reference gene. Data represent relative mRNA expression of **(A)** *Erp27*, **(B)** *CPA5*, **(C)** *CPA1*, **(D)** *CTRL*, **(E)** *LOC109066737*, **(F)** *ssbp4*, **(G)** *NEU3*, and **(H)** *Per2* determined by quantitative real-time PCR.

The real-time PCR results were consistent with the RNA-seq data (Figure 5). Eight transcripts were selected, and the RNA-seq data were further evaluated by real-time PCR experiments. The relative expression levels of carboxypeptidase A1 (*CPA1*), carboxypeptidase A5 (*CPA5*), chymotrypsin-like (*CTRL*), and endoplasmic reticulum resident protein 27-like (*Erp27*) were significantly increased in the 6.35 Trp group compared to those in the control group. In addition, the relative expression levels of period circadian protein homolog 1-like (*LOC109066737*), neuraminidase 3 (*NEU3*), period circadian regulator 2 (*Per2)*, and single-stranded DNA binding protein 4 (*ssbp4*) were significantly decreased in the 6.35 Trp group compared to the control group.

### Gut Microbial Composition

**Figure 6:**
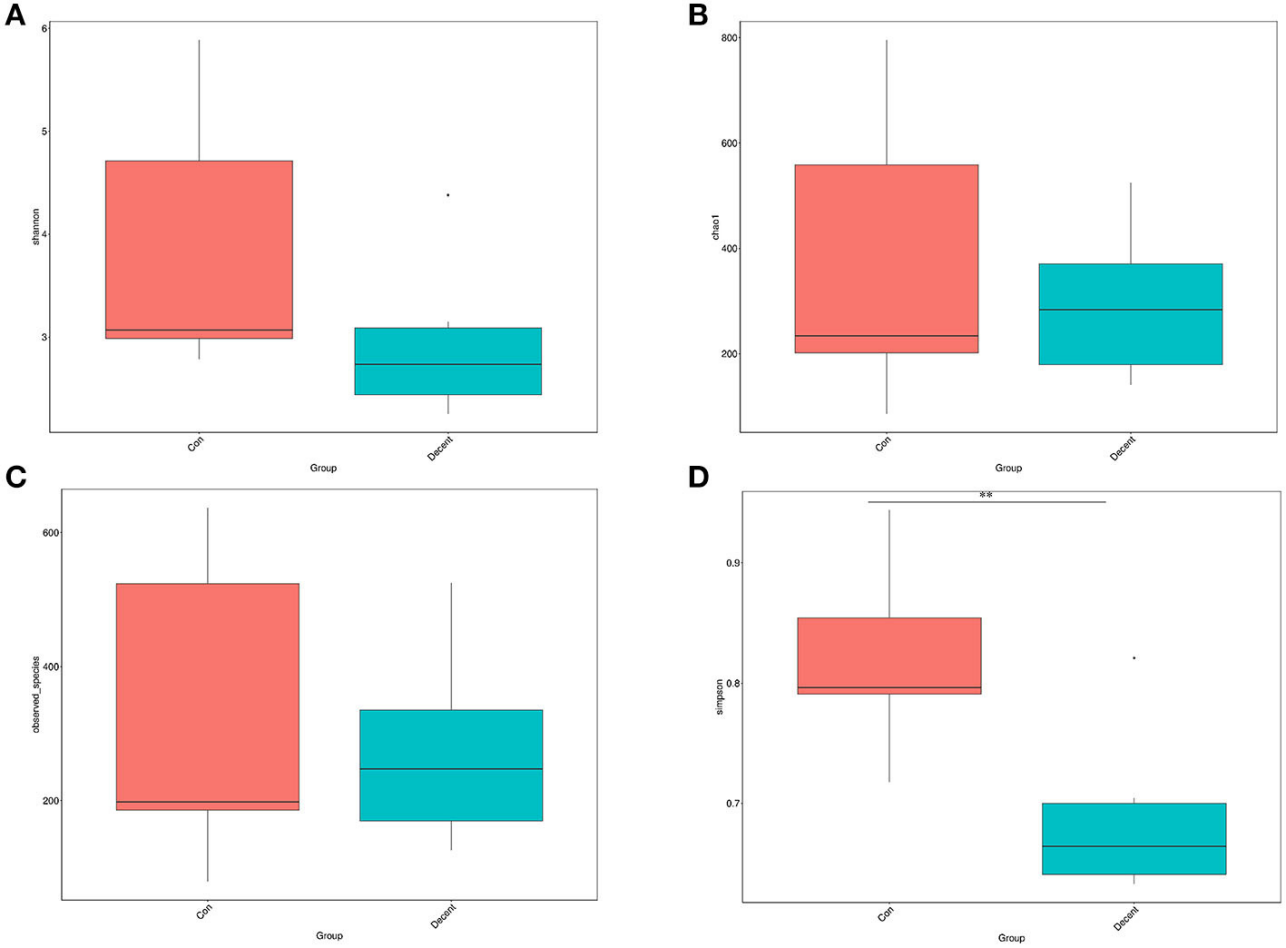
Alpha diversity of the gut microbiota in control group and 6.35Trp group. **(A)** Shannon's diversity index. **(B)** Chao1. **(C)** Observed-species. **(D)** Simpson. **p < 0.01 vs. control group by Tukey's *post-hoc* test.

**Figure 7:**
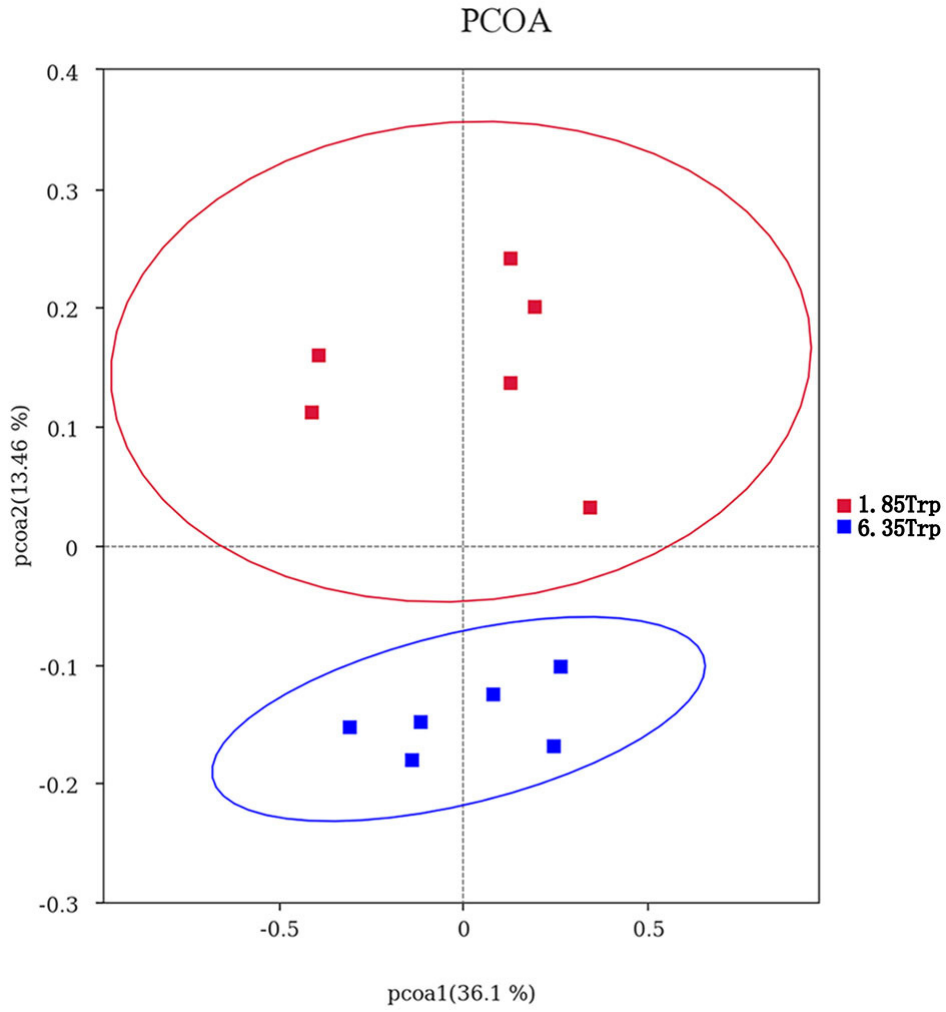
Principal coordinate analysis (PCoA) of the microbial community composition of the control group and 6.35 Trp group. Horizontal coordinates indicate one principal component, vertical coordinates indicate another principal component, and percentages indicate the value of the principal component's contribution to sample differences; each point in the graph represents a sample, and samples from the same group are represented using the same color. Blue represents the 6.35 Trp group, and red represents the 1.85 Trp group.

OTUs were defined as a read sharing 97% nucleotide-sequence identity (Supplementary Table 7). The number of observed species and indices of Shannon, Simpson, and Chao1 did not differ between the 6.35 Trp and control groups (Figure 6). Compared to the control group, the Simpson index (*P* < 0.05) in the 6.35 Trp group was decreased significantly, which indicated that the bacterial diversity was increased after 6.35 g kg^−1^ Trp diets treatment. PCoA is a comparative analysis of the microbial community composition of different samples (Figure 7). The unweighted UniFrac distance-based PcoA results showed that the microbiota compositions of the control group and the 6.35 Trp group were overt changes.

**Figure 8:**
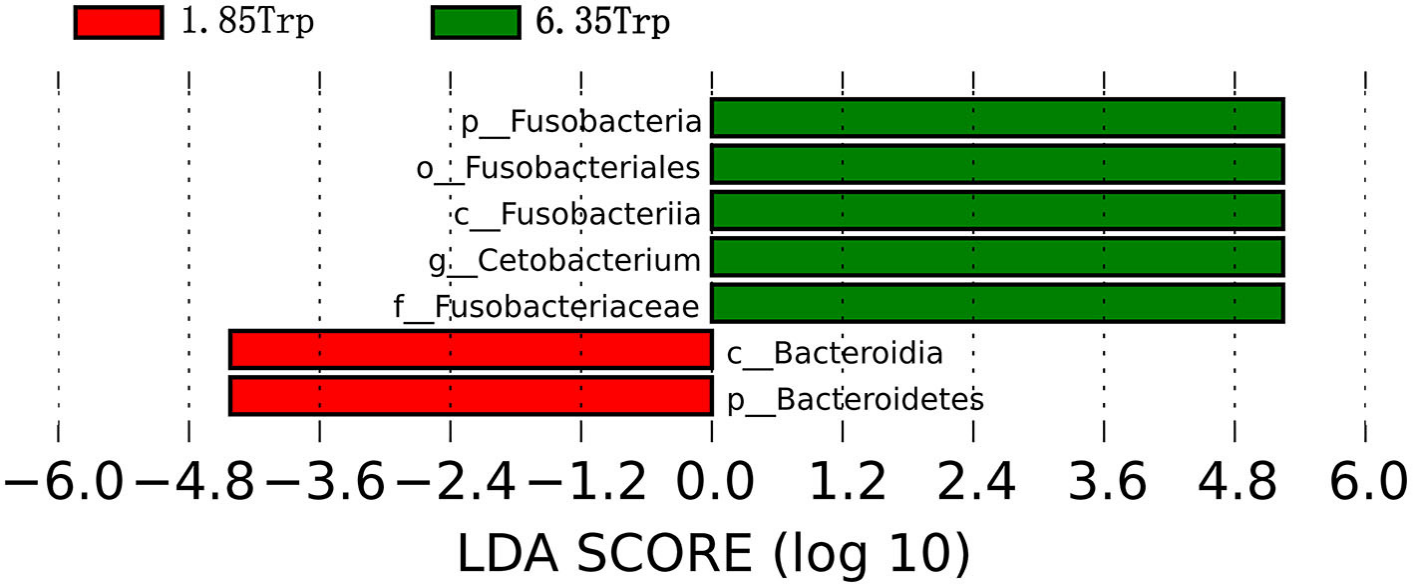
Linear discriminant analysis coupled with effect size (LEfSe) of the control group and 6.35 Trp group. The LDA value distribution histogram shows species with an LDA score greater than the set value (set to 4 by default), and the biomarkers with statistically significant differences between groups. The prefixes “p,” “c,” “o,” “f,” “g,” “s,” and “t” represent the annotated level of phylum, class, order, family, genus, species, and strain. Green represents the 6.35 Trp group, and red represents the 1.85 Trp group.

To identify the significantly different species of gut microbes between the control and 6.35 Trp groups, we analyzed the microbial community profiles using LEfSe software (Figure 8). There were seven significantly different biomarker, with enrichment of *p_Fusobacteria, o_Fusobacteriales, c_Fusobacteria, g_cetobacterium*, and *f_Fusobacteriaceae* in the 6.35 Trp group and *c_Bacteroidia*, and *c_Bacteroidetes*, in the control group. At the genus level, compared to the control group, Cetobacterium was significantly more abundant in the 6.35 Trp group (*P* < 0.05). At the phylum level, the abundance of *Fusobacteria* increased, but the abundance of *Proteobacteria* decreased in the 6.35 Trp group. The Firmicutes /B acteroidetes ratio was increased compared to that of the control group (*P* < 0.05).

## Dicussion

### Growth Performance

Trp, an essential amino acid, is important for metabolic functions in fish (30–32). Some Trp metabolites are also important mediators to regulate partial physiological functions in fish (4). Previous studies have reported that different dietary levels of Trp affect growth performance in many fish, including juvenile silver catfish, and fingerling Indian catfish (11, 33). Trp improved hybrid catfish growth performance, digestive, and absorptive abilities (34). In this study, we found that the growth performance of ITCC increased as the dietary Trp levels increased (up to 6.35 g kg^−1^). This result supports that optimal dietary Trp could improve the growth performance and feed efficiency as reported in juvenile Jian carp (*Cyprinus carpio* var. Jian) (13), silver catfish (33), red drum (14), Indian catfish (Ahmed et al., 2012), and Nile tilapia (35). Analogously, increases in the dietary Trp concentrations can promote the ADG, and a certain dose-dependent relationship has been found between dietary Trp and the growth performance of fishes (36–40). However, another study showed that dietary supplementation with Trp had no effects on the growth performance and body proximate of seabream (Sparus aurata) (41). A possible reason for this discrepancy may be related to ethnic differences and different diets.

### Trp Improved the Intestinal Morphology of ITCC

The integrity of the intestine is important for nutrient uptake and intestinal health (42–44). Generally, digestion and absorption depend on intestinal growth and development, as well as the activities of digestive enzymes in fish. The VH and CD are important indices of the functional capacity of enterocytes, and the VH: CD ratio affects the nutrient digestibility and absorption capacity of the intestinal mucosa (45, 46). The improvement of intestinal morphology was associated with increased nutrient absorption and growth performance of fish (47). Previous studies have demonstrated that fish have a special need for Trp in epithelial structures. Optimal Trp exerts beneficial effects on maintaining the intestinal structural integrity and intestinal development of fish (13, 48). In this study, feeding 6.35 g kg^−1^ Trp diets significantly increased villus height and crypt depth, which suggests that dietary Trp can influence the morphological structure of the intestine, which might be associated with nutrient digestion and absorption. Similar results showed that Trp improves the digestive and absorption capacity of fish (34, 49).

### Trp Regulated the Expression of Genes in the Intestine of ITCC

Regarding the molecular mechanism by which Trp affects intestinal morphology, pathway enrichment of DEGs summarizes the complex networks of genes.

#### Fatty Acid Biosynthesis

Fatty acid biosynthesis capacity of fish varies among species, with trophic level hypothesized as a major factor (50). Fatty acid catabolism is a major source of energy in salmonid fish (51). In the present study, 6.35 g kg^−1^ Trp gut samples showed an upregulated expression of genes enriched significantly in Fatty acid biosynthesis signaling pathway (*ACS*L, *FADD, ACACA*, and *FASN*) compared with control group. This indicated that 6.35 g kg^−1^ Trp intake further promoted fatty acid biosynthesis in ITCC. Acetyl-CoA carboxylase (*ACACA*) and fatty acid synthase (*FASN*) are important rate-limiting enzymes that play a critical role in body weight differences in abdominal adipose tissue of growing animals (52). *FASN* plays a crucial role in the process from lipogenesis and is physiologically modulated by energy balance (53). So, the expression of *FASN* may be a good non-invasive indicator to study the role of Trp in growth and in studies on fatty acid biosynthesis in ITCC. Therefore, studying the regulation of *Fatty acid biosynthesis* has great significance for improving gut health.

#### Protein Digestion and Absorption

Fish growth is based on the digestion and absorption of nutrients (54). Fish are known to utilize proteins preferentially to lipids or carbohydrates as energy sources (55). Amino acids are important energy sources, satisfying 14–85% of the energy needs of teleost fish (54). A previous study showed that dietary Trp could improve the digestion and absorption ability of juvenile Jian carp (*Cyprinus carpio var*. Jian) (13). In this work, numerous genes related to “protein digestion and absorption” were upregulated in the intestine of fish fed 6.35 g kg^−1^ Trp diets, indicating that dietary Trp might influence associated with changed intestinal function in protein digestion and absorption of ITCC.

#### AMPK Signaling Pathway

*TOR* can activate the AMPK signaling pathway and control the growth response of cells to nutrients, especially amino acids (56). *TOR* is a nutrient sensor that can affect cell growth by regulating of protein synthesis (57, 58). Previous studies have reported that dietary Trp supplementation increased the expression of *TOR* and *S6K1* mRNA levels and the phosphorylation level of *TOR* and *S6K1* in grass carp muscle (59, 60). In this work, dietary Trp levels (6.35 g kg^−1^ Trp diet) upregulated the relative expression of *TOR* mRNA in the intestinal tract, in agreement with the finding that dietary Trp increased the expression of *TOR* in hybrid catfish (34). Conversely, another study showed that dietary Trp improved young grass carp growth, which may be related to the downregulation of *TOR* in the intestine of young grass carp (36). These results suggest that Trp may activate the AMPK signaling pathway to coordinate nutrient uptake in fish through regulation of *TOR* gene expression. However, the mechanisms require further study.

### Intestinal Microorganism and Tryptophan

Trp plays important roles in maintaining gut microflora and intestinal health. Deficiency in dietary Trp could alter the gut microbial community (61). In this study, we found that 6.35 g kg^−1^ Trp significantly increased the abundance of *Cetobacterium*, and the Firmicutes/Bacteroidetes ratio at the phylum level (*P* < 0.05). A study by Liang et al. reported that Trp can increase intestinal species richness (62). Studies have shown that the ratio of Firmicutes/Bacteroidetes is related to increased energy harvesting and growth performance (63–65). Similarly, it was found that the higher the ratio of the relative abundance of *Firmicutes* to *Bacteroidetes* in grass carp, the faster the growth of the fish (66). It is worth noting that Trp increased the richness and diversity of the intestinal microbiota, perhaps partly because Trp promoted the growth of the intestinal villi, thus increasing the nutrients available to the intestinal flora (34, 62, 67).

Moreover, *Cetobacterium* has been identified as an important component of gut microbiota in freshwater fishes, which is an indicator of healthy fish (68–71). A previous study found the effect of diet on the abundance of *Cetobacterium* in the intestine of zebrafish (72). Numerous studies have confirmed the effect of *Cetobacterium* on the digestion and absorption of food, and the general growth and development process of fishes (Yunlong Grouper, common carp, and tilapia) (73, 74). In the present study, the abundance of *Cetobacterium* was increased significantly by 6.35 g kg^−1^ Trp treatment in ITCC, suggesting that *Cetobacterium* might play a crucial role in digestive and nutritional processes.

In addition, it was also found that in the 6.35 Trp group, the abundance of *Fusobacteria* increased after dietary with 6.35 g kg^−1^ Trp. *Fusobacteria* is the most dominant phylum in the fish intestine and it may have a lasting positive impact on intestinal function (17, 75). *Fusobacteria* may be involved in the digestive process of fish by providing a variety of enzymes (76). Previous studies have shown that Trp catabolites are absorbed through the intestinal epithelium and enter the bloodstream, affecting host physiology and promoting intestinal and systemic homeostasis (31). Thus, the increase in the abundance of *Cetobacterium* and *Fusobacteria* might indicate the positive effect of Trp in balancing the gut microbiota, which may be most strongly linked to health performance.

Overall, our study suggests that dietary 6.35 g kg^−1^ Trp had a beneficial effect on gut microbes and regulated the abundance of gut microbes in ITCC. However, further work needs to be done determine the effects of Trp on certain beneficial intestinal bacteria.

## Conclusions

In this study, dietary Trp was found to improve the growth performance and intestinal health of ITCC. We found that 6.35 g kg^−1^ Trp altered intestinal gene expression involved in protein digestion and absorption and the AMPK signaling pathway in the ITCC gut. In addition, 6.35 g kg^−1^ Trp significantly increased the abundance of *Cetobacterium* and the Firmicutes/Bacteroidetes ratio in the ITCC gut. However, more studies are needed to clarify the interaction between host gene expression and gut microbiota in ITCC fed a diet with 6.35 g kg^−1^ Trp.

## Data Availability Statement

The raw reads were deposited to Sequence Read Archive (SRA) database (PRJNA702642 for RNA-seq; BioProject: PRJNA704527 for 16S rRNA sequencing).

## Ethics Statement

The animal study was reviewed and approved by the Animal Welfare Committee of the Institute of Subtropical Agriculture (201703-64C), Chinese Academy of Sciences, Changsha, China.

## Author Contributions

KX and FH conceptualized and designed this study. YF, XL, DL, HG, YW, and WL performed the main experiments and analyzed the data. FH participated in experimental animal management, tissue sampling, and data analysis. YF, XL, and DL drafted this manuscript. KX and FH reviewed this manuscript. KX acquired the funding and supervised this study. All authors read and approved the manuscript.

## Conflict of Interest

The authors declare that the research was conducted in the absence of any commercial or financial relationships that could be construed as a potential conflict of interest.
