# Supplementary material for: Effect of Dietary Tryptophan on Growth, Intestinal Microbiota, and Intestinal Gene Expression in an Improved Triploid Crucian Carp
Source: Front Nutr. 2021 Jun 17;8:676035. doi: 10.3389/fnut.2021.676035 (PMC8247481; doi:10.3389/fnut.2021.676035)
Supplement: Supplementary file 6 [file Table_6.docx]

| Catelogy | Rank methods in cytoHubba | | | | | | | |
| --- | --- | --- | --- | --- | --- | --- | --- | --- |
|  | MCC | MNC | Degree | EPC | Closeness | | Radiality |  |
| Gene symbol top  15 | ubr4 | skp2 | ubr4 | hecw2a | ubr4 | cbl | |  |
|  | skp2 | socs3a | skp2 | HERC1 | cbl | waslb | |  |
|  | fbxl14a | fbxl14a | kbtbd8 | ube2q2 | alcama | LOC561737 | |  |
|  | fbxo30a | fbxo30a | socs3a | herc2 | skp2 | reps1 | |  |
|  | kbtbd8 | kbtbd8 | fbxl14a | skp2 | waslb | alcama | |  |
|  | plzfa | plzfa | fbxo30a | fbxl14a | kbtbd8 | fynb | |  |
|  | socs3a | trip12 | plzfa | cdc34b | trip12 | actr2a | |  |
|  | hecw2a | ube3c | trip12 | ube3c | socs3a | actr2b | |  |
|  | HERC1 | cdc34b | LTN1 | uba1 | fbxl14a | arrb2b | |  |
|  | ube2q2 | uba1 | ube3c | ube2kb | fbxo30a | trioa | |  |
|  | herc2 | hectd1 | cdc34b | trip12 | plzfa | igf2r | |  |
|  | cdc34b | hecw2a | uba1 | ubr4 | hectd1 | cltcb | |  |
|  | LTN1 | HERC1 | hectd1 | hectd1 | ube3c | ap2a1 | |  |
|  | ube3c | ube2q2 | alcama | alcama | LTN1 | hip1 | |  |
|  | uba1 | herc2 | hecw2a | fbxo30a | LOC561737 | agfg1a | |  |

Supplementary Table6. Hub genes ranked by the Degree method in cytoHubba
